# Small and Equipped: the Rich Repertoire of Antibiotic Resistance Genes in Candidate Phyla Radiation Genomes

**DOI:** 10.1128/mSystems.00898-21

**Published:** 2021-12-07

**Authors:** Mohamad Maatouk, Ahmad Ibrahim, Jean-Marc Rolain, Vicky Merhej, Fadi Bittar

**Affiliations:** a Aix-Marseille Univ, IRD, APHM, MEPHI, Marseille, France; b IHU Méditerranée Infection, Marseille, France; Institute of Urban Environment, Chinese Academy of Sciences

**Keywords:** CPR, antibiotic resistance, antimicrobial classes, microbial war, glycopeptide, beta-lactam, aminoglycoside

## Abstract

Microbes belonging to Candidate Phyla Radiation (CPR) have joined the tree of life as a new branch, thanks to the intensive application of metagenomics and sequencing technologies. CPR have been eventually identified by 16S rRNA analysis, and they represent more than 26% of microbial diversity. Despite their ultrasmall size, reduced genome, and metabolic pathways which mainly depend on exosymbiotic or exoparasitic relationships with the bacterial host, CPR microbes were found to be abundant in almost all environments. They can be considered survivors in highly competitive circumstances within microbial communities. However, their defense mechanisms and phenotypic characteristic remain poorly explored. Here, we conducted a thorough *in silico* analysis on 4,062 CPR genomes to search for antibiotic resistance (AR)-like enzymes using BLASTp and functional domain predictions against an exhaustive consensus AR database and conserved domain database (CDD), respectively. Our findings showed that a rich reservoir of divergent AR-like genes (*n* = 30,545 hits, mean = 7.5 hits/genome [0 to 41]) were distributed across the 13 CPR superphyla. These AR-like genes encode 89 different enzymes that are associated with 14 different chemical classes of antimicrobials. Most hits found (93.6%) were linked to glycopeptide, beta-lactam, macrolide-lincosamide-streptogramin (MLS), tetracycline, and aminoglycoside resistance. Moreover, two AR profiles were discerned for the *Microgenomates* group and “*Candidatus* Parcubacteria,” which were distinct between them and differed from all other CPR superphyla. CPR cells seem to be active players during microbial competitive interactions; they are well equipped for microbial combat in different habitats, which ensures their natural survival and continued existence.

**IMPORTANCE** To our knowledge, this study is one of the few studies that characterize the defense systems in the CPR group and describes the first repertoire of antibiotic resistance (AR) genes. The use of a BLAST approach with lenient criteria followed by a careful examination of the functional domains has yielded a variety of enzymes that mainly give three different mechanisms of action of resistance. Our genome analysis showed the existence of a rich reservoir of CPR resistome, which is associated with different antibiotic families. Moreover, this analysis revealed the hidden face of the reduced-genome CPR, particularly their weaponry with AR genes. These data suggest that CPR are competitive players in the microbial war, and they can be distinguished by specific AR profiles.

## INTRODUCTION

The increased use of exploring tools in the 21st century, such as high-throughput sequencing and its wide application in metagenomics, has led to broadening access to genomic data of uncultured microorganisms (1). These previously unknown genomes have challenged the classical view of the tree of life and have given rise to new divisions (2). Representatives of these divisions have been moved out of the group of undiscovered living organisms (microbial dark matter) (3). Among these discoveries, many questions have been raised about a new group of microbes which is close to bacteria but quite unique and known as Candidate Phyla Radiation (CPR) (4, 5).

CPR are a group of highly distinct and abundant ultrasmall microbes (6, 7), which represent more than 26% of known bacterial diversity (3). These microbes are characterized by their reduced-size genomes (8) and the occurrence of a high percentage of unknown-function proteins (9). Recently, a comparative study of protein families between CPR and bacteria showed that CPR have a prevalence of proteins involved in a symbiotic lifestyle and interaction with other microbes (9, 10). This interaction is required, since they have minimal metabolic capabilities (11). Therefore, they are highly auxotrophic with a lack of essential encoding genes for some pathways which are critical to the autonomous lifestyle (12).

Paradoxically, the lack of these genes can sometimes help them to survive in their habitat (13). For example, despite the absence of a viral CRISPR defense system in *Patescibacteria* (the superphylum that contains most CPR genomes), members of this superphylum can escape bacteriophage attacks (attachment) by the natural suppression of common phage membrane receptors (14).

In addition, these newly described microbes are considered uncultured bacteria to date. They have been detected thanks to metagenomic or metabarcoding analyses of their rRNA sequences (4). However, few members of the *Saccharibacteria* superphylum have been isolated in coculture with an obligatory bacterial host (15). This association is essential for its viability and growth (15). The first cocultured *Saccharibacteria* strain (TM7x, “*Candidatus* Nanosynbacter lyticus”) was cocultured in 2015 based on its streptomycin resistance selection (15). So far, a few members of this superphylum have been successfully cocultured with different bacterial hosts by different protocols (15–17).

To date, only three CPR superphyla (*Saccharibacteria*, *Gracilibacteria*, and *Absconditabacteria*) have been reported in different human microbiomes (for example, buccal cavity, gut microbiota, vagina, etc.) (11, 13, 18–22). In addition to these groups, all CPR members are abundant in the environment (for example, soil, seawater, deep-sea sediments, termite guts, etc.) (23–28). Their ubiquitous presence in complex ecosystems therefore suggests their continuous competitive lifestyle against different microorganisms. This focuses attention on understanding the defensive mechanisms employed by CPR microbes in habitats shared with other microbes.

Moreover, according to metagenomic analyses of ancient DNA, CPR microbes have been reported in ancient samples of Neanderthal calcified dental plaque (calculus) dated thousands of years ago (29). In parallel, survival strategies, including antibiotic resistance (AR) gene components, have long been reported in the microbial world (30, 31). Various studies have shown the natural existence of AR genes in microorganisms even before the discovery and introduction of antibiotics by humans in the mid-20th century (32). These AR genes have also been detected from ancient samples dating back millions of years in diverse environments (32). The mechanisms of AR are due to the absence of antibiotic targets, their modification following a mutation on preexisting genes, or the presence of protein-coding genes (33). Some genes can inactivate the antibiotic by enzymatic activity, while other genes confer AR by target protection or alteration (33).

Given that CPR members (i) are widely spread in different ecological niches and microbiomes, (ii) have never been isolated and grown in pure culture, and (iii) have a high number of unknown biosynthetic activities within their genomes, few if any studies have investigated the defense mechanisms and competing behavior of CPR cells. In fact, survival strategies, which are, namely, AR gene components expressed by CPR members against other microbes in different hostile/competitive environments, have not yet been explored. For this purpose, we describe for the first time the first repertoire of AR genes in CPR genomes by *in silico* analysis using BLAST and search for functional domains. We found that CPR members are also players in this microbial “infinity war.”

## RESULTS

### CPR microbes contain vastly divergent AR-like genes according to reference bacterial protein databases.

**FIG 1:**
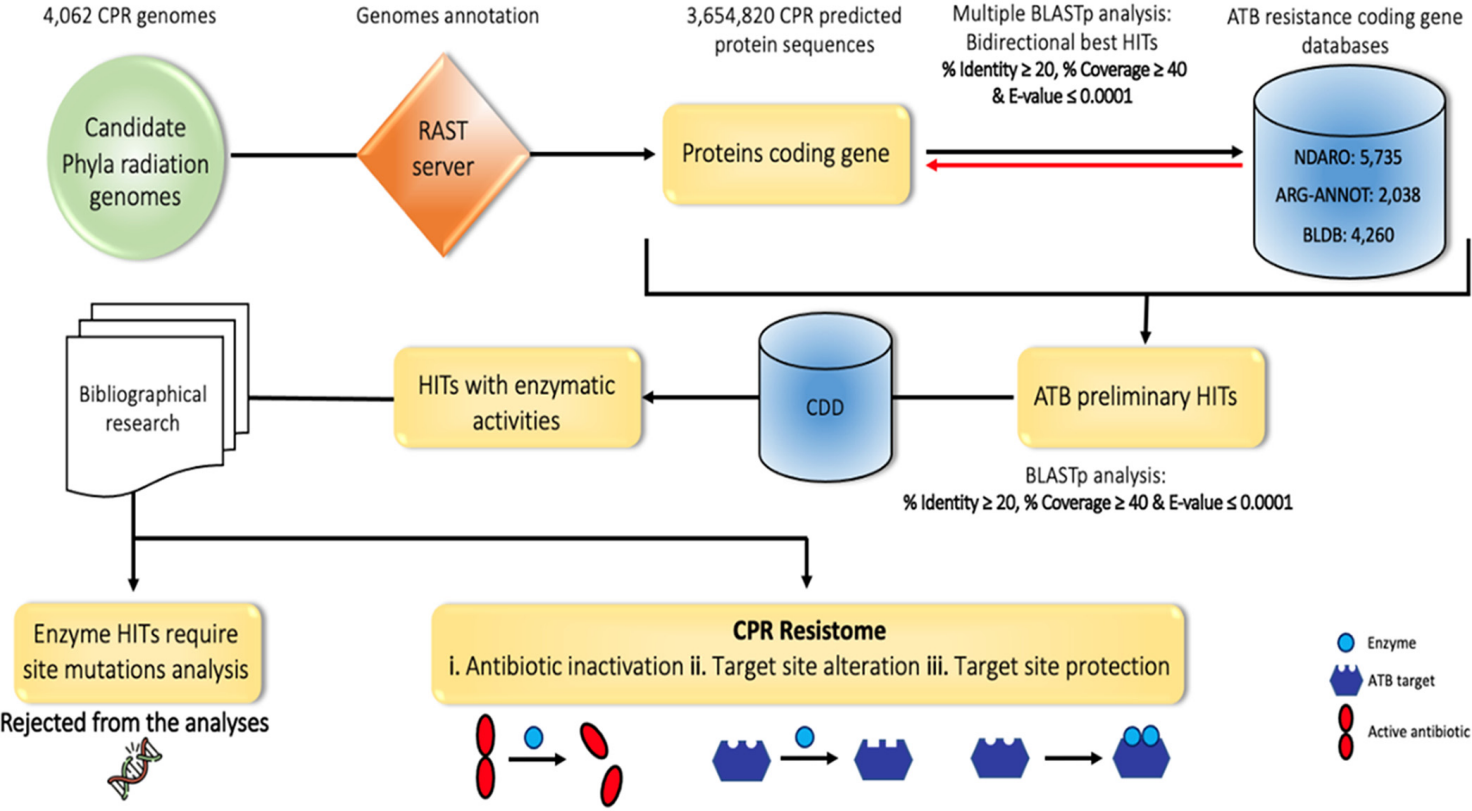
Study design. The first step consists of annotating the CPR genomes available on the NCBI website, using the RAST server. The CPR protein sequences are considered queries for BLASTp against consensus databases of bacterial antibiotic resistance (AR) genes. The analysis was performed with a minimum identity and coverage percentage of 20% and 40%, respectively, and a maximum E value of 0.0001. The AR preliminary hits resulting from the simple BLASTp are queried against the multiple databases of AR genes as performing a reciprocal BLASTp. Further analyses were undertaken to detect the protein functional domain for hits with enzymatic activity using the conserved domain database (CDD). Finally, bibliographical research was conducted to select enzymes conferring resistance with specific mechanisms of actions as CPR resistome.

**FIG 2:**
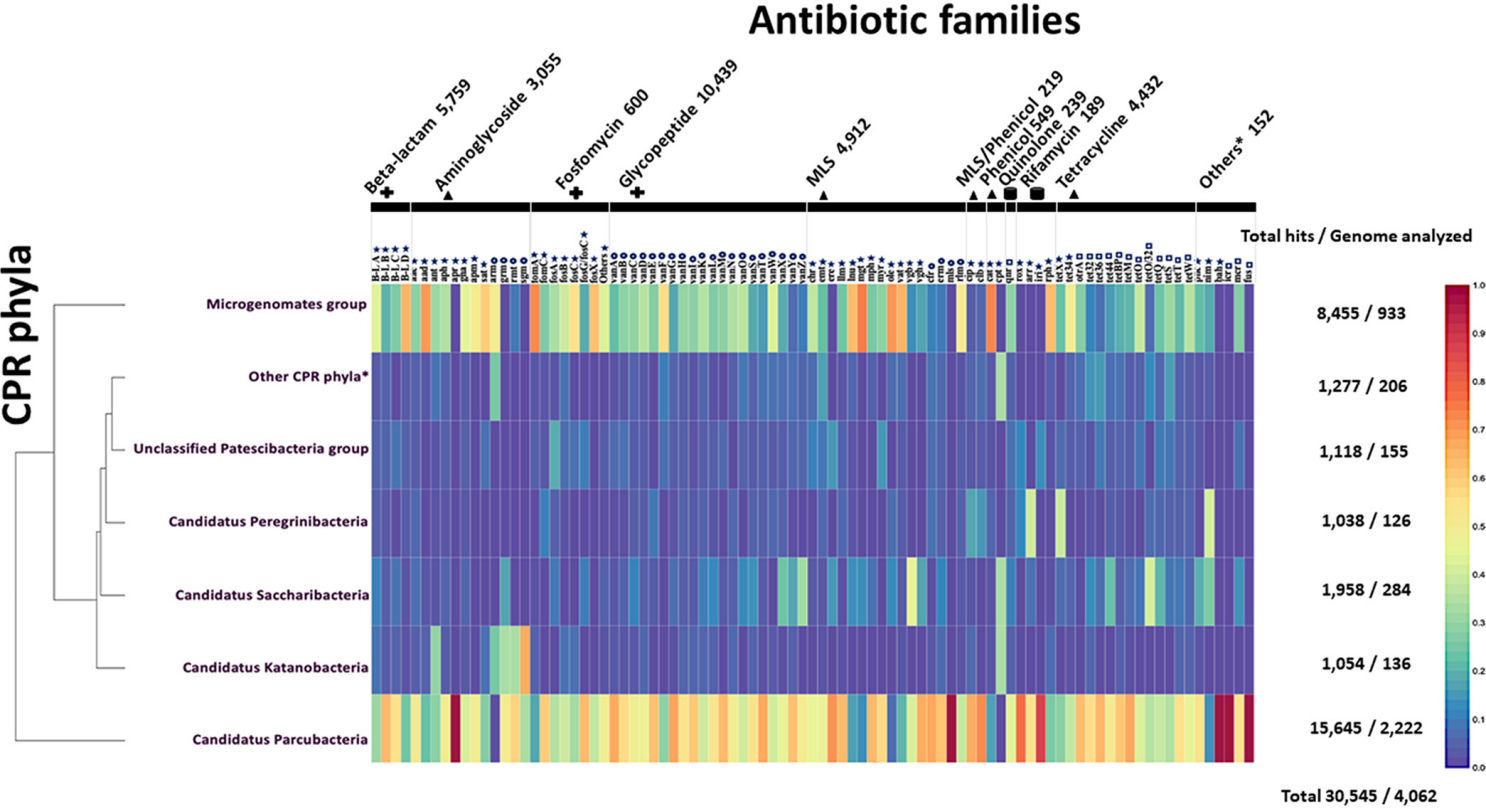
Multi-informative heat map of antibiotic resistance (AR)-like genes in CPR genomes. Detection of 30,545 AR-like genes in 4,062 CPR genomes using an adapted AR screening strategy. The abundance of each AR-like gene on each CPR phylum is relative to the total number of AR-like genes found in all CPR phyla (number of AR-like genes found in CPR phylum divided by the total hit number of this AR family). MLS indicates the merging of the three antibiotic families macrolide, lincosamide, and streptogramin. “Others*” indicates the merging of five antibiotic families with fewer AR-like genes: pyrazinamide, nitroimidazole, bacitracin, colistin, and fusidic acid. A cross indicates an antibiotic that acts on the cell wall, a filled triangle indicates an antibiotic that acts on the ribosome, and a filled square indicates an antibiotic that acts on the nucleic acid. A star indicates AR-like genes that confer resistance by antibiotic-inactivating enzymes, an open circle indicates AR-like genes that confer resistance by antibiotic target alteration, and an open square indicates AR-like genes that confer resistance by antibiotic target protection. “Other CPR phyla*” indicates the merging of all “*Candidatus*” CPR phyla with fewer than 100 genomes: “*Candidatus* Berkelbacteria,” “*Candidatus* Doudnabacteria,” “*Candidatus* Wirthbacteria,” Candidate division Kazan, “*Candidatus* Dojkabacteria,” “*Candidatus* Absconditabacteria,” and “*Candidatus* Gracilibacteria.” The cladogram of CPR superphyla was based on the clustering of the heat map.

In this study, we developed an adaptive strategy for the specific detection of AR-like genes in the 4,062 CPR genomes tested (Fig. 1; see also Table S1 in the supplemental material) and assigned them according to the available taxonomy of NCBI into 10 CPR superphyla. Only the superphylum *Patescibacteria* was divided into 4 groups or phyla: *Parcubacteria*, *Gracilibacteria*, *Microgenomates*, and unclassified *Patescibacteria*. The simple BLASTp search of the 3,654,820 CPR protein sequences predicted using the RAST server, against a total of 12,033 AR protein sequences, resulted in 320,121 matches (Fig. 1). Reciprocal BLASTp followed by the search for conserved protein functional domains against the conserved domain database (CDD) led to 175,238 potential AR genes (Fig. 1; see also Materials and Methods). We then focused only on enzyme-encoding genes that confer resistance to a given antibiotic family. However, after eliminating all BLASTp matches (hits) that need further examination of mutations (134,693 hits), we could retain a total of 30,545 hits (Fig. 2; Tables S2 and S3), corresponding to 89 AR-like genes (Fig. 2; Tables S2 and S3). These genes constituted the target data set in our analysis and were considered the CPR resistome (Fig. 1). This is used for deciphering the high potential of proto-resistance genes as a deep reservoir of AR in these microorganisms.

10.1128/mSystems.00898-21.5 TABLE S1 Table showing data for the CPR genomes tested in this study. Download Table S1, XLSX file, 0.3 MB. Copyright © 2021 Maatouk et al. 2021 Maatouk et al. https://creativecommons.org/licenses/by/4.0/ This content is distributed under the terms of the Creative Commons Attribution 4.0 International license.

10.1128/mSystems.00898-21.6 TABLE S2 Metadata table containing the CPR genomes analyzed and their AR-like gene contents. Download Table S2, XLSX file, 1.9 MB. Copyright © 2021 Maatouk et al. 2021 Maatouk et al. https://creativecommons.org/licenses/by/4.0/ This content is distributed under the terms of the Creative Commons Attribution 4.0 International license.

10.1128/mSystems.00898-21.7 TABLE S3 Number of antibiotic resistance hits according to CPR superphyla. Download Table S3, XLSX file, 0.02 MB. Copyright © 2021 Maatouk et al. 2021 Maatouk et al. https://creativecommons.org/licenses/by/4.0/ This content is distributed under the terms of the Creative Commons Attribution 4.0 International license.

**FIG 3:**
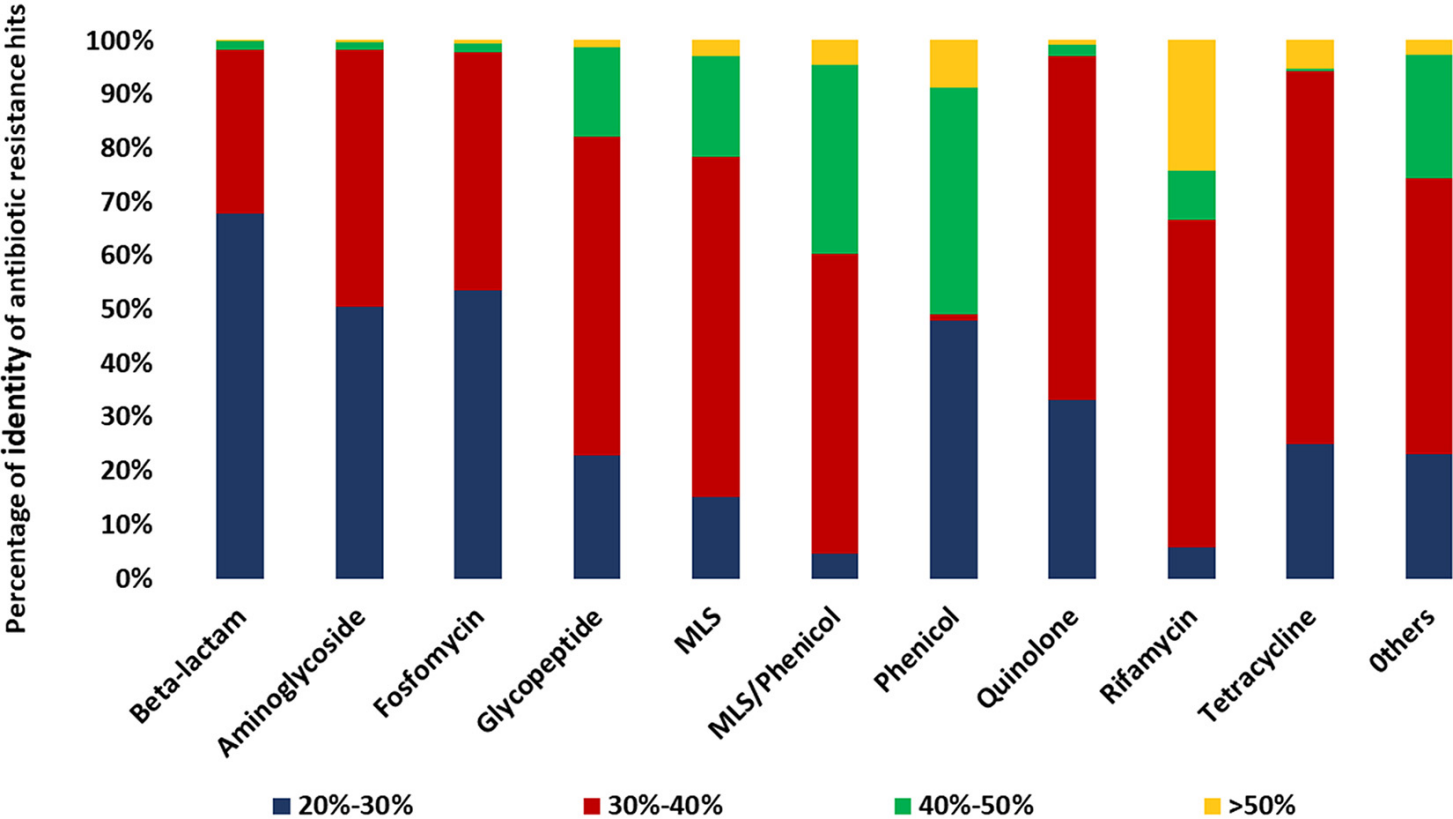
Histogram representing the distribution of antibiotic-resistant (AR) hits by percentage of similarity against bacterial AR genes in each antibiotic family detected in this study. “Others” indicates the merging of five antibiotic families with fewer AR-like genes: pyrazinamide, nitroimidazole, bacitracin, colistin, and fusidic acid.

Even though these potential resistance genes in CPR microbes had the same functional domains as bacteria, most AR gene sequences found in CPR had a similarity percentage ranging from 30% to 40% with bacterial AR gene sequences (Fig. 3). This highlights the divergence of the CPR sequences from those of bacteria. High divergence of CPR sequences was observed specifically in beta-lactam, aminoglycoside, fosfomycin, and phenicol antibiotic resistance families with low percentages of sequence similarity (20% to 30%) with those of the bacteria (Fig. 3). It is noteworthy that among the total of 30,545 detected AR-like genes, 10,443 were annotated as hypothetical proteins by the RAST server despite having the functional domain responsible for the resistance to a proper antibiotic family.

**FIG 4:**
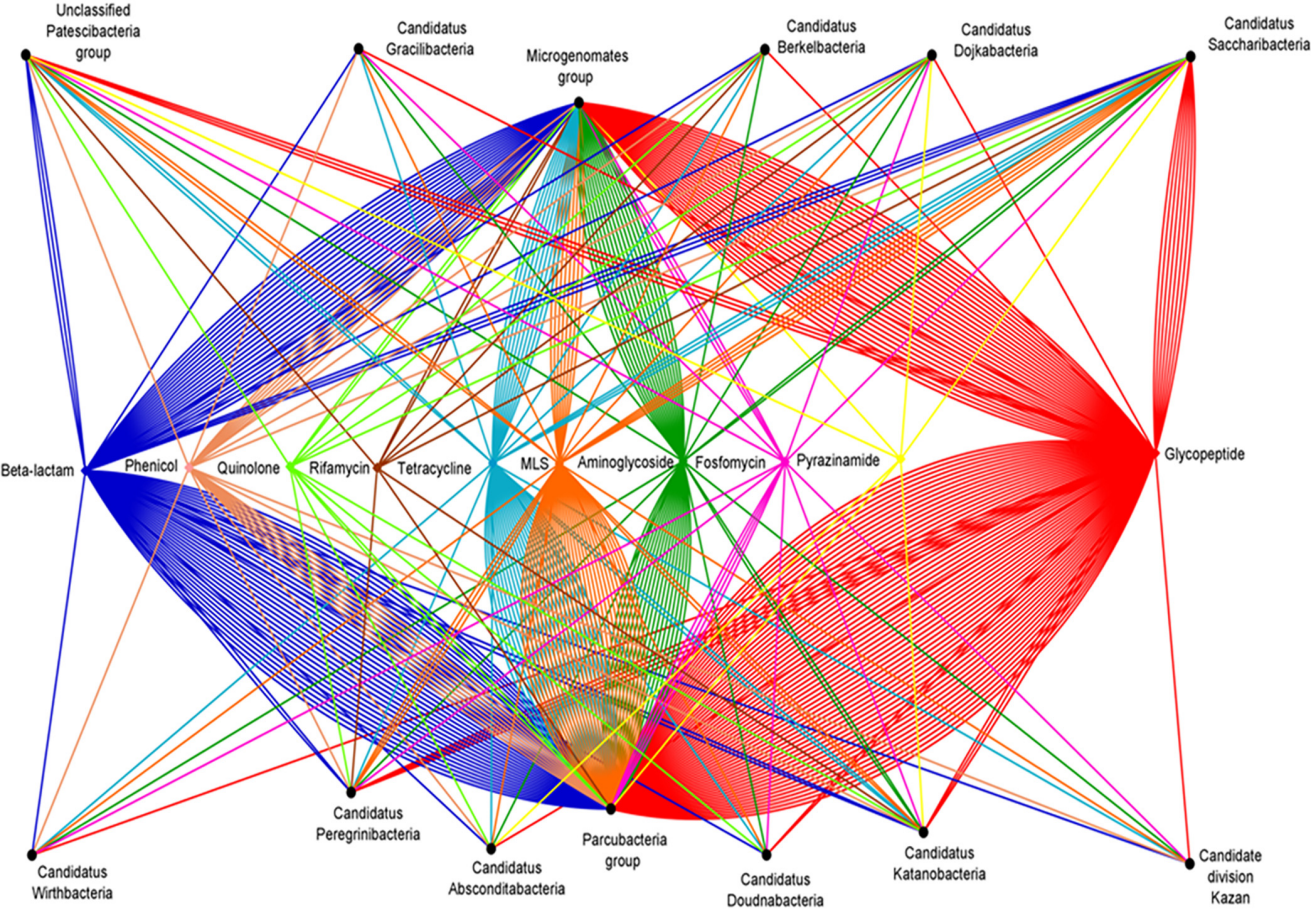
Network analysis of antibiotic resistance-like gene distribution, highlighting the link between different antibiotic families and distinct CPR superphyla. Beta-lactam in blue, phenicol in rose, quinolone in light green, rifamycin in brown, tetracycline in light blue, MLS in orange, aminoglycoside in dark green, fosfomycin in pink, pyrazinamide in yellow, and glycopeptide in red.

The CPR resistome showed high diversity, as it could be associated with 14 different antibiotic families: 34.18% glycopeptide, 18.85% beta-lactam, 10% aminoglycoside, 14.51% tetracycline, 16.08% macrolide-lincosamide-streptogramin (MLS), 1.8% phenicol, 1.96% fosfomycin, 0.62% rifamycin, 0.78% quinolone, and 0.5% other antibiotic families (bacitracin, fusidic acid, pyrazinamide, nitroimidazole, and lipopeptides) (Fig. 2 and 4; see also Tables S2 and S3). A high percentage of the AR hits identified in our study confer AR by altering the antibiotic’s target, with methyltransferase activity of 16S rRNA (47.34% of total hits: 14,459 hits), whereas others act directly on a given antibiotic by inactivating it (37.73% of total hits: 11,525 hits) or by protecting its target (14.93% of total hits: 4,561 hits) (Fig. 2 and 4; see also Tables S2 and S3).

**FIG 5:**
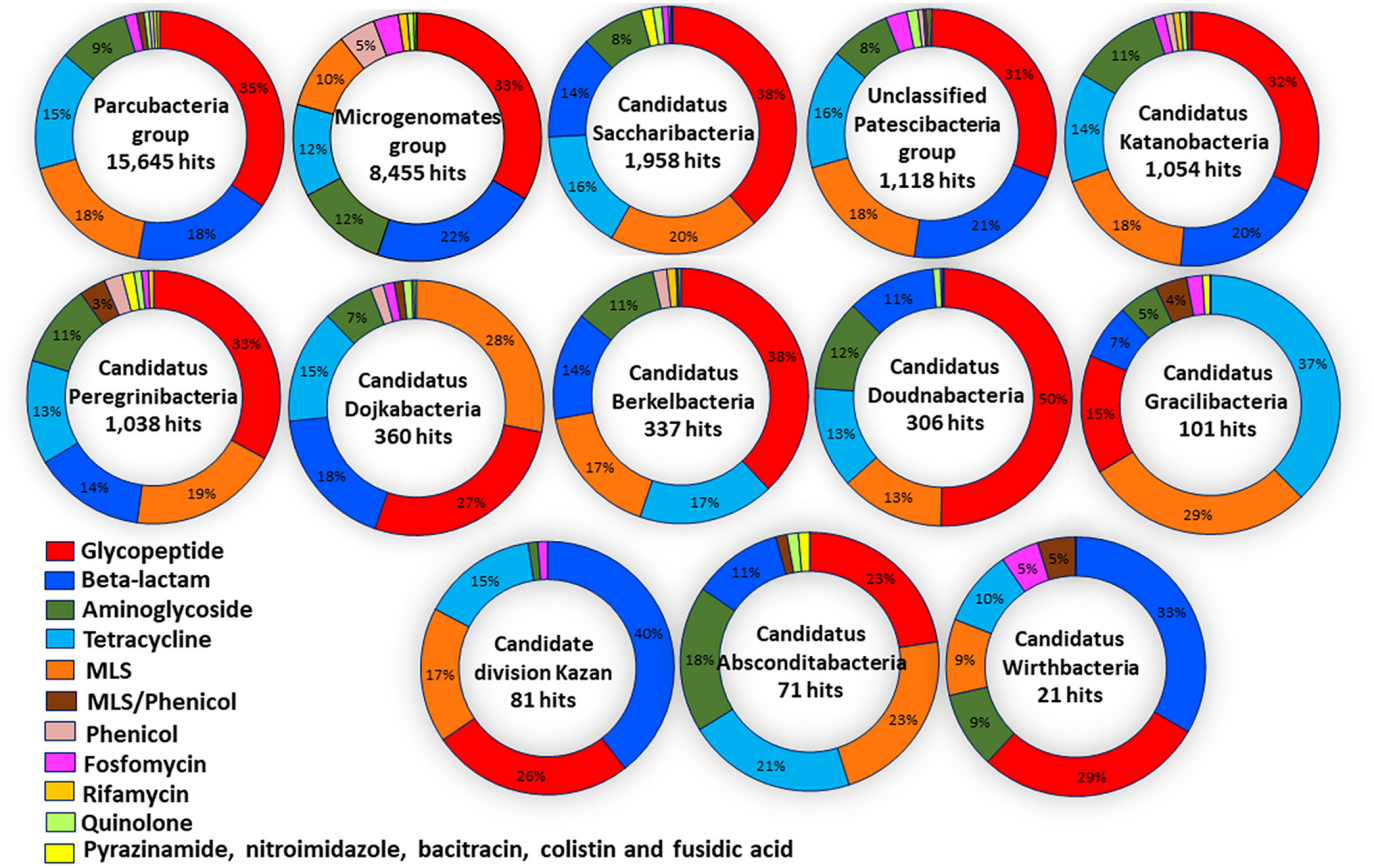
The distribution of the percentage of antibiotic resistance-like genes by antibiotic families on each CPR superphylum.

In addition, we found AR hits in almost all studied CPR genomes across different superphyla; thus, 4,052 genomes were positive through our analysis out of 4,062 genomes tested (99.75%). The prevalence of the AR content was fairly diversified between the CPR superphyla, as the number of their available genomes was not homogeneous (Fig. 2 and 4; see also Tables S2 and S3). Furthermore, each CPR superphylum holds AR genes to at least six different classes of antibiotics, and they have nearly the same distribution of AR hits (Fig. 5). The different CPR superphyla had in common AR genes to five antibiotic families, namely, glycopeptide, beta-lactam, MLS, tetracycline, and aminoglycoside, which highlight the importance of the function of these AR hits in CPR genomes.

### The prevalence of detected enzymes according to each chemical antibiotic class.

In this part, we examined all chemical classes of antibiotics for which we have detected hits in all CPR superphyla. Starting with glycopeptide, the resistance hits were found to be the most abundant AR-like genes. Glycopeptide antibiotics destabilize the cell wall by interfering with peptidoglycan synthesis (34). Resistance to glycopeptide (particularly vancomycin) involves the modification of the antibiotic target d-alanine:d-alanine into d-alanine:d-lactate or d-alanine:d-serine (34). Since vancomycin resistance is mediated by a cluster of genes including essential, regulatory, and accessory genes, we searched within CPR genomes for the presence of at least the three essential genes in the cluster (35). These essential genes can be classified into nine sorts based on their genetic sequences and structures: *vanA*, *vanB*, *vanC*, *vanD*, *vanE*, *vanG*, *vanL*, *vanM*, and *vanN* (35).

Forty-eight of the CPR genomes have a potential for vancomycin resistance as they carry the three essential genes for the functioning of a given cluster. We looked for the gene that gives the cluster name, plus *vanH* and *vanX* for d-Ala:d-Lac clusters and *vanX* and *vanT* for d-Ala:d-Ser clusters. We found a total of 18 d-Ala:d-Lac vancomycin clusters, including nine *vanA* clusters, three *vanB* clusters, five *vanD* clusters, and one *vanM* cluster. For d-Ala:d-Ser ligase gene clusters, we found 25 *vanC* clusters, one *vanE* cluster, three *vanG* clusters, three *vanL* clusters, and two *vanN* clusters (a total of 34 d-Ala:d-Serine vancomycin clusters) (Fig. S1). Of these 48 genomes, four had two different types of vancomycin clusters: one genome presented the essential genes of the *vanB* and *vanD* clusters, one genome had *vanA* and *vanC* clusters, and two genomes had *vanL* and *vanN* clusters (Fig. S1). More analysis is needed to search for the presence of other components (such as regulatory and accessory genes) and to verify the synteny of these genes as they participate together in the correct functioning of the vancomycin cluster.

10.1128/mSystems.00898-21.1 FIG S1 Heat map of different vancomycin clusters d-Alanine:d-Lactate and d-Alanine:d-Serine with the presence of the three essential genes in the CPR superphyla tested. Forty-eight CPR genomes have a total of 52 potential vancomycin clusters including four genomes each with two types of clusters. Download FIG S1, PDF file, 0.1 MB. Copyright © 2021 Maatouk et al. 2021 Maatouk et al. https://creativecommons.org/licenses/by/4.0/ This content is distributed under the terms of the Creative Commons Attribution 4.0 International license.

Given that CPR members have very small genomes in comparison with other microorganisms (2, 4), it is profitable for these microbes to have multifunctional genes, such as beta-lactamases (36). The beta-lactam-resistant enzymes (namely, beta-lactamase) hydrolyze the beta-lactam ring in their molecular structure (37). The 5,759 beta-lactam-resistant hits belong to four different classes (A, B, C, and D) (Fig. 2; see also Tables S2 and S3). Class B metallo-beta-lactamases are the most frequent, representing 58.3% of those detected (3,359 hits over 5,759 hits) (Tables S2 and S3). This class has been classified into three different subclasses of metallo-beta-lactamases depending on the annotation of the CDD results: 17 hits belong to subclass B1, 385 hits to subclass B2, and 2,957 hits to subclass B3. Moreover, 2,400 of the serine-beta-lactamases are distributed over 27.9% of class A, 0.5% of class C, and 13.3% of class D (Tables S2 and S3).

Although macrolide-lincosamide-streptogramin (MLS) antibiotics are chemically distinct, but because of their similar mechanism of action, they are classified in the same group (38). MLS antibiotics act on the 50S subunit of the 23S rRNA gene (39, 40). The most common genes (*erm* [*n* = 3,077 hits] and *cfr* [*n* = 648 hits]) (Fig. 2; see also Tables S2 and S3) detected in the CPR genomes are involved in MLS resistance by altering the MLS target with esterase activity and methylation of the 23S rRNA subunit, respectively, followed by streptogramin acetyltransferase (*vat*; *n* = 338 hits) with MLS-inactivating enzyme activity (Tables S2 and S3). In addition, our study showed aminoglycoside resistance hits in all CPR superphyla with different transferase activities including adenylyltransferase, phosphotransferase, and acetyltransferase. Aminoglycosides are a family of molecules containing an aminocyclitol ring where they bind to the A site of the ribosome and disrupt protein synthesis (41). The majority of aminoglycoside-resistant-like genes code for acetyltransferase activity, of which the most abundant genes are *aac* (aminoglycoside acetyltransferase, *n* = 1,831) and *gna* (gentamicin acetyltransferase, *n* = 749) (Fig. 2; Tables S2 and S3). Finally, almost all tetracycline-resistant hits confer resistance through ribosomal protection. The genes encoding tetracycline resistance ribosomal protection protein in the CPR were *tetT* (*n* = 2,243), *tetBP* (*n* = 778), and *tetW* (*n* = 564) (Fig. 2; Tables S2 and S3).

### Antibiotic resistance profile according to CPR superphyla.

Based on our AR screening strategy (Fig. 1), only 10 genomes out of the 4,062 analyzed genomes were found to not contain AR genes. The other genomes were found to be positive with a notable average of 7.5 AR-like genes per genome. The general distribution of hits classed according to antibiotic family was almost consistent in the various CPR superphyla, with some exceptions (Fig. 5), despite the high difference in the number of AR hits found between the *Parcubacteria* phylum including most CPR genomes and “*Candidatus* Wirthbacteria” with only two genomes (15,645 AR hits in 2,222 tested genomes compared to 21 AR hits, respectively) (Fig. 2, 4, and 5; see also Tables S2 and S3). Interestingly, CPR superphyla were clustered into three major groups according to their AR content and the abundance of the detected genes (Fig. 2; see also Fig. S2). The first group includes *Parcubacteria* genomes, the second includes *Microgenomates* genomes, and the last group includes the remaining CPR superphyla. Three different AR profiles were therefore identified for CPR superphyla. In the *Microgenomates* group, we observed a significant number of genes with adenylyltransferase (*aad*) and acetyltransferase (*gna*) activity against aminoglycosides, phosphorylation of fosfomycin (*fomA*), and a remarkable number of class A and D beta-lactamases (Tables S2 and S3). Moreover, this group of *Microgenomates* possesses the greatest number of *cat* (chloramphenicol acetyltransferase) enzyme-encoding genes, streptogramin acetyltransferase (*vat*) genes, and rifampin phosphotransferase (*rph*) genes detected among all CPR genomes. In contrast, taking *Saccharibacteria* as an example of the group of other CPR superphyla, members of this superphylum have a high number of streptogramin lyases (*vgb*) and erythromycin esterases (*erm*) compared to other CPR groups (Fig. 2; see also Tables S2 and S3).

10.1128/mSystems.00898-21.2 FIG S2 Multi-informative heat map (Fig. 4) with the grouping of “*Candidatus* Saccharibacteria,” unclassified *Patescibacteria* group, Candidate division WWE3 (*Katanobacteria*), “*Candidatus* Peregrinibacteria,” “*Candidatus* Berkelbacteria,” “*Candidatus* Dojkabacteria,” “*Candidatus* Doudnabacteria,” “*Candidatus* Gracilibacteria,” “*Candidatus* Absconditabacteria,” Candidate division Kazan-3B-28, and “*Candidatus* Wirthbacteria” together as the other CPR group. Download FIG S2, PDF file, 0.5 MB. Copyright © 2021 Maatouk et al. 2021 Maatouk et al. https://creativecommons.org/licenses/by/4.0/ This content is distributed under the terms of the Creative Commons Attribution 4.0 International license.

**FIG 6:**
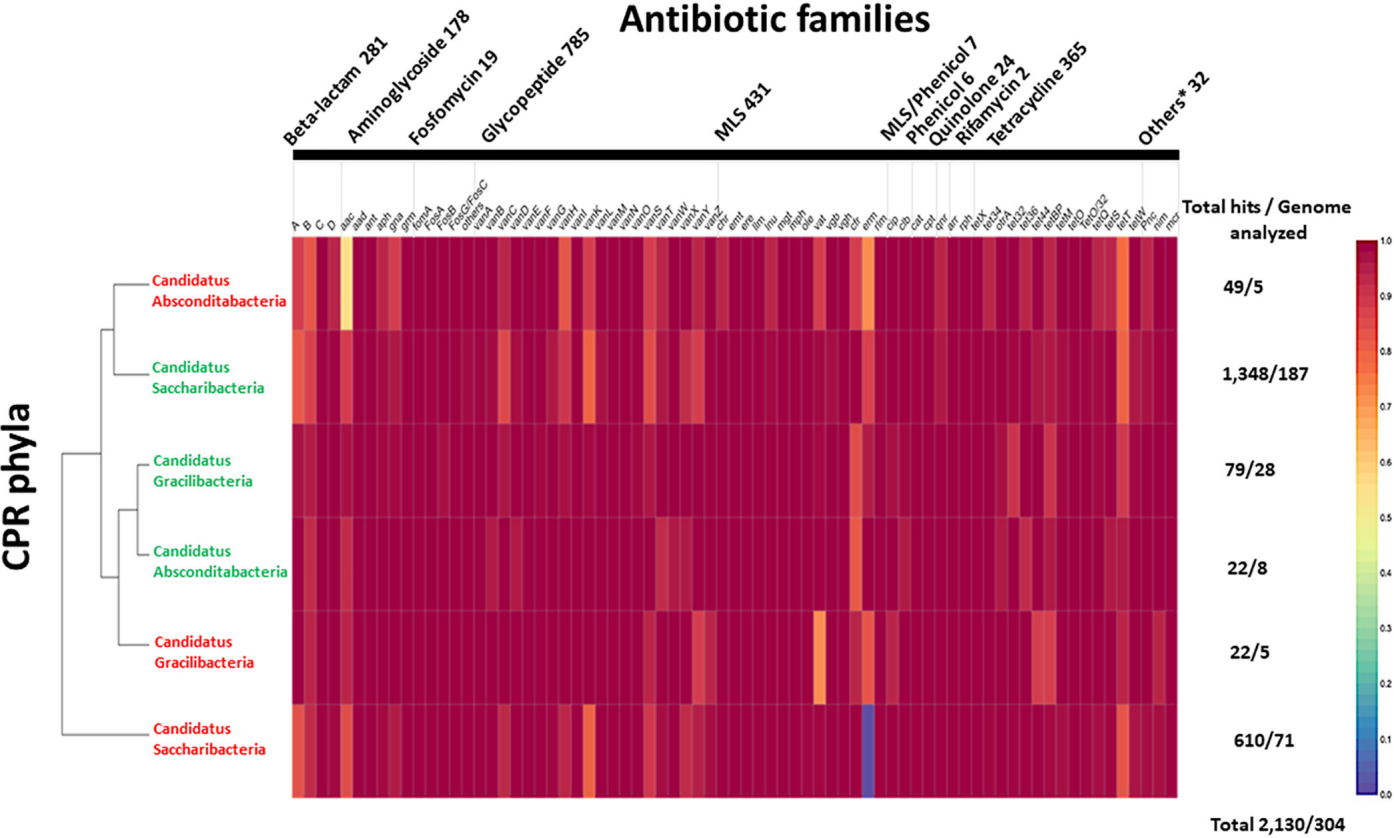
Heat map of antibiotic resistance-like genes in the genomes of the 3 CPR superphyla detected in humans (red) and the environment (green). The heat map was generated by dividing the number of AR hits by the number of tested genomes in each superphylum (human versus environment). “Others*” indicates the merging of three antibiotic families: pyrazinamide, nitroimidazole, and colistin. The cladogram of CPR superphyla was based on the clustering of the heat map.

It should be noted that the more available genomes we analyzed, the more likely we were to detect additional antibiotic resistance families. This is the case for the *Parcubacteria* group, where *bah* (the amidohydrolase enzyme that inactivates bacitracin), *icr* (intrinsic colistin resistance enzyme), and *fus* (fusidic acid resistance enzyme) were found only in this CPR group (Fig. 2; see also Tables S2 and S3). Finally, considering the isolation source (i.e., human-associated genomes versus environmental genomes) for a given CPR lineage, each of the three CPR superphyla found regularly in humans, namely, *Saccharibacteria*, *Gracilibacteria*, and *Absconditabacteria*, showed a quantitative variability aspect of AR-like gene content compared to their environmental counterparts (Fig. 6; Fig. S3). In the *Saccharibacteria* superphylum, for example, the *erm* gene is more abundant in human-associated CPR genomes (258 hits over 71 genomes) than in those found in the environment (80 hits over 187 genomes) (Fig. 6; Table S4). It is also the case for the *vat* gene in the *Gracilibacteria* phylum (5 hits for human-associated CPR genomes and 1 hit for others recovered from environmental sources). Interestingly, genomes from the three human-associated CPR superphyla showed a higher average of AR-like genes per genome than others detected from the environment. This was found specifically in the *Absconditabacteria* superphylum, with 9.8 AR-like genes/genome for those detected in humans and 2.75 for others found in the environment (Fig. 6; see also Table S4).

10.1128/mSystems.00898-21.3 FIG S3 Network analysis of antibiotic resistance-like gene distribution between CPR genomes detected in humans (red) and the environment (green). (A) *Saccharibacteria* superphylum. (B) *Absconditabacteria* superphylum. (C) *Gracilibacteria* phylum. Download FIG S3, PDF file, 0.1 MB. Copyright © 2021 Maatouk et al. 2021 Maatouk et al. https://creativecommons.org/licenses/by/4.0/ This content is distributed under the terms of the Creative Commons Attribution 4.0 International license.

10.1128/mSystems.00898-21.8 TABLE S4 Number of antibiotic resistance hits according to the 3 CPR superphyla found in human and environmental samples. Download Table S4, XLSX file, 0.01 MB. Copyright © 2021 Maatouk et al. 2021 Maatouk et al. https://creativecommons.org/licenses/by/4.0/ This content is distributed under the terms of the Creative Commons Attribution 4.0 International license.

To sum up, these results are suggestive of the influence of the CPR environment on its phenotypic characteristics and suggest a link between CPR members, other microbes, and their environment. Together, the high presence of AR-like genes in all CPR genomes may suggest that they have other vital functions for CPR cells and that therefore they are not strictly related to antibiotic resistance. They are likely to be functionally linked to other metabolic pathways and, subsequently, to participate in the survival of these microorganisms.

## DISCUSSION

There are significant knowledge gaps in our understanding of the physiological and biological processes of CPR, as well as of their interactions with host bacteria and their potential associations with human pathologies. Thus, it is essential to expand our research on these living microorganisms, which represent a new branch in the tree of life (2). This study aimed to report the existence of AR in these microbes and to determine the AR profile of each CPR superphylum. These analyses may contribute toward a better elucidation of CPR phenotypic characteristics and defense mechanisms.

Here, we conducted a thorough *in silico* screening for AR in all CPR genomes available on NCBI, regardless of their quality and assembling methods, to detect the maximum of AR genes. The superphylum assignments were taken from NCBI and were not independently confirmed in our study, since our main aim is not reclassifying CPR but reporting AR genes in all their available genomes. Our analysis was based on a thoughtful strategy for these new microorganisms, using computational methods with adapted criteria. We revealed a rich repertoire of AR genes contained in almost all tested CPR genomes. We allocated the AR-like genes into families/groups to visualize the prevalence of AR genes in different CPR superphyla and, potentially, to find a correlation between genes encoding resistance to a particular antibiotic family and the superphylum of interest.

Since resistance has never been searched for in CPR before and given that CPR microbes have not yet been grown in pure culture without their bacterial host, their resistance can be explored only by *in silico* analysis for the moment. AR screening of CPR genomes by analyzing nucleotide sequences against a database of bacterial resistance genes (the classical method of AR profiling in the bacterial domain) (42) resulted in a negligible number of hits compared with our optimized strategy. Only five CPR genomes were positive with a total of 9 AR genes. These genes fall within the AR genes found using our optimized strategy with protein sequences of the CPR genomes tested (see Table S5 in the supplemental material).

10.1128/mSystems.00898-21.9 TABLE S5 Number of antibiotic resistance (AR) hits according to all CPR genomes tested, using the default parameters of ABRicate and ARG-ANNOT as AR database. Download Table S5, XLSX file, 0.01 MB. Copyright © 2021 Maatouk et al. 2021 Maatouk et al. https://creativecommons.org/licenses/by/4.0/ This content is distributed under the terms of the Creative Commons Attribution 4.0 International license.

It was critical to establish an adapted strategy for AR screening in CPR genomes, as they have different nucleotide and protein sequences from bacterial ones (1). Because of the functional constraints, protein sequences have a low evolutionary rate compared to DNA sequences (43). Accordingly, we used the protein sequences that were determined by RAST annotation, which gave the low percentages of unannotated proteins (44). The high percentage of hypothetical proteins obtained is concentrated in CPR, because many of their metabolic pathways and biosynthetic capacities have not been determined yet (10).

Attempting to study a new branch of the tree of life when there is a huge lack of data is challenging. For this reason, less stringent BLAST parameters were used to achieve a more comprehensive exploration of the AR contents (9). Multiple AR gene databases were used to detect maximum hits, since there is currently no specific AR database for CPR members. In addition, a reciprocal BLASTp search was performed to reduce the number of false-positive results. Then, we identified the functional protein domains for the detected hits to retain only the protein sequences with patterns related directly to the AR and more precisely the enzymatic activities. These enzymes confer resistance by acting directly on the inactivation of the corresponding antibiotic or by protection or alteration of its target. The AR genes that require small nucleotide polymorphism to generate resistance were discarded from the analyses since CPR sequences are neither comparable nor similar to those of bacteria. To benchmark our AR screening strategy, we did the same analysis against a well-characterized set of conventional bacteria [Streptomyces coelicolor A3(2) and Streptomyces hygroscopicus] (Fig. S4). Interestingly, after keeping only the enzyme-encoding genes containing AR domains (33 and 37 AR-like genes, respectively), we had very similar results as the RAST annotation (according to PATRIC) of the 2 tested genomes (35 and 34 AR genes, respectively) (Fig. S4). In summary, our strategy and specifically the screening of AR domains give reliable results and can help to characterize new AR genes even in bacteria (45). Altogether, our multistep study design guarantees an optimal balance between the intended function (specificity) and permissive stringency (sensitivity).

10.1128/mSystems.00898-21.4 FIG S4 Antibiotic resistance-like genes detected in the *Streptomyces* genomes using the adapted screening strategy of CPR and according to PATRIC with RAST server annotation. Download FIG S4, PDF file, 0.05 MB. Copyright © 2021 Maatouk et al. 2021 Maatouk et al. https://creativecommons.org/licenses/by/4.0/ This content is distributed under the terms of the Creative Commons Attribution 4.0 International license.

Nevertheless, despite our precautions to be as exhaustive as possible, this strategy might have missed some resistance genes, leading to false-negative results. It could be expected that CPR members have AR sequences that are significantly different from those of bacteria, with new patterns and undescribed resistance mechanisms. Even though they may have evolved from within bacteria or have emerged from an unknown protogenote with bacteria (9), they definitely have sequences divergent from those of bacteria due to rapid evolutionary phenomena (1). Indeed, the AR genes of CPR were not found to be very similar to the AR genes in the AR databases from bacteria. In addition to the resistance profiling found in this study, the possible presence of efflux pumps in CPR cells, as in all living microorganisms, which participate in the detoxification process by expelling various harmful and xenobiotic compounds, should not be overlooked. In particular, these include the multidrug efflux mechanisms which are normally encoded by the chromosome (46).

The surprising and somewhat paradoxical presence of AR-like genes in the reduced genomes of CPR raises questions about their origin and their real function in these microorganisms. These genes can give CPR advantages to resist antibiotics released by other microorganisms sharing a common ecological niche with them. Thanks to our strategy, 34.2% of the results (10,344 out of 30,545 AR-like genes) can be now reannotated as potential AR genes instead of being simple hypothetical proteins. The divergence observed in these sequences suggests that they have other functions involved in different metabolic pathways rather than resistance to antibiotics. Further studies are needed to confirm their functions.

Concerning the resistance to the glycopeptide family, we could detect 20 different types of vancomycin resistance, in the 4,062 CPR genomes, which covers the high diversity that has previously been described for these genes (35). However, because the function of these genes depends on their presence in an operon (33), the lack of synteny compared to the better-characterized vancomycin clusters seems to bring into question the effectiveness of this system. At first glance, it seems important to search for the additional components, including accessory and regulatory genes, to have a complete vancomycin cluster (33).

However, as described previously, the membrane of CPR cells is very similar to that of Gram-positive bacteria (9), which develop resistance to vancomycin by modifying the d-alanine:d-alanine peptidoglycan precursor (33). CPR microbes seem to have a natural presence of regulatory genes in their genomes; thus, efflux pumps like *vanR* are present in all CPR genomes (100%) (data not shown). These genomes may naturally produce the d-Ala:d-Lac or d-Ala:d-Ser peptidoglycan precursors rather than the natural precursor d-Ala:d-Ala in bacteria. This supports the intelligent way in which these microorganisms survive with a limited number of genes, that is, an incomplete but functional cluster (i.e., no need for accessory genes, as their name indicates). This supports the idea that the CPR genome is simple but efficient. Further analysis should be carried out to verify the AR conferred by the absence or modification of the antibiotic’s targets, in addition to that conferred by the presence of active enzymes determined as part of this study.

Our results also show that there is almost one beta-lactam resistance gene per CPR genome; 77% of the tested genomes have at least one gene which codes for beta-lactamases (classes A, B, C, and D). These genes may play a role in the degradation of substances used in metabolic pathways, including beta-lactams. Several studies have shown that beta-lactamase genes are multifunctional genes which play several roles including, but not limited to, endonuclease, exonuclease, RNase, and hydrolase (47). Furthermore, beta-lactamases have been detected in other life domains including bacteria (48), eukaryotes (49), and archaea (50) and therefore may also be present in CPR. It is very likely that the presence of multifunctional genes is necessary and indispensable in CPR members, due to their small genomes and the very reduced number of genes per genome compared to other microorganisms.

Interestingly, aminoglycoside resistance has been mentioned and used for the coculture of TM7x (a phylotype of “*Candidatus* Saccharibacteria”) with its host species bacterium, Schaalia odontolytica strain XH001 (15). The authors enriched TM7x through streptomycin selection, as its host is also highly resistant to streptomycin. It is likely that CPR members are resistant to aminoglycosides and other antibiotics targeting RNA. Besides having an uncommon ribosome composition/sequence, some CPR have introns in their 16S and 23S rRNA and tRNA (15). Given their tiny genomes, this is a prominent feature for them to carry multifunctional genes, depending on the intron splitting.

It is worth noting that the number of AR-like genes was not found to be correlated with genome size. Thus, the CPR that are found in the environment have a larger genome size but a smaller average of AR-like genes than the CPR detected in human microbiomes. This reversed tendency in the three CPR superphyla (*Saccharibacteria*, *Gracilibacteria*, and *Absconditabacteria*) detected in humans and the environment can be linked to the source of isolation of CPR genomes tested and/or to antibiotic consumption in humans.

The significant prevalence of AR genes in this new branch of the tree of life sheds light on the problem of choosing the appropriate treatment in the clinical field. The overlooking of AR screening in CPR might be responsible for the observed failure to provide adequate antibiotic treatment. It is important to investigate whether this failure of different cases is due to the presence of hidden resistance genes or the presence of resistance genes that have not been searched for. Indeed, our study has already confirmed that CPR genomes can act as resistance vectors that can transfer the AR profile to bacteria even without needing gene transfer events. Consequently, these AR genes may also give advantages to the attached bacteria (host bacteria) to survive in the environment (which secrete antibiotic without having the convenable resistance gene) or against other microbes which produce antibiotics. Otherwise, CPR could use their AR profile to protect their bacterial host against a given antibiotic, since they cannot survive without a viable one.

Finally, the AR-like genes detected in CPR genomes in our *in silico* screening are expected to be confirmed in upcoming *in vitro* experiments. A specific database for AR gene screening in CPR genomes needs to be created to collect these new results for further studies.

To conclude, this work contributes toward a new way of deciphering this new branch of the tree of life. We explicitly explored the CPR resistome by establishing an adapted AR screening strategy for these fastidious microorganisms. We found a gigantic reservoir of provisional AR, representing the first report of resistance genes in CPR genomes. These highly abundant microbes could be an interesting paradigm which constitutes an endless natural source of emerging resistances. Our findings represent a substantial opportunity for future scientific discoveries. If, as expected, the AR-like genes detected in CPR are involved in different metabolic pathways, further studies may lead to the successful growth of CPR cells in pure culture.

## MATERIALS AND METHODS

### Genomic data.

For this study, all nucleotide sequences of CPR genomes available on 12 September 2020 on the NCBI website (National Center for Biotechnology Information) (https://www.ncbi.nlm.nih.gov) were selected and downloaded from the NCBI GenBank database (see Table S1 in the supplemental material). Genomes were chosen based on the taxonomy provided by the NCBI (Table S1). The 4,062 CPR genomes are distributed across 2,222 “*Candidatus* Parcubacteria,” 933 “*Candidatus* Microgenomates,” 284 “*Candidatus* Saccharibacteria,” 155 unclassified *Patescibacteria* group, 136 Candidate division WWE3 (*Katanobacteria*), 126 “*Candidatus* Peregrinibacteria,” 55 “*Candidatus* Berkelbacteria,” 53 “*Candidatus* Dojkabacteria,” 39 “*Candidatus* Doudnabacteria,” 33 “*Candidatus* Gracilibacteria,” 13 “*Candidatus* Absconditabacteria,” 11 Candidate division Kazan-3B-28, and two “*Candidatus* Wirthbacteria” (under reserve of NCBI superphylum assignments) (Table S1). Only 35 of all the genomes analyzed were complete genomes, while the remaining were whole-genome sequences (WGS). In addition, 2 bacterial genomes of *Streptomyces* spp. [Streptomyces coelicolor A3(2) with GenBank ID CP042324.1 and Streptomyces hygroscopicus with GenBank ID CP003275.1] were selected and downloaded from the NCBI GenBank database.

Genome annotation was generated using the Rapid Annotation using Subsystem Technology tool kit (RASTtk) as implemented in the PATRIC v3.6.8 annotation web service (51) (Fig. 1).

### Detection of antibiotic-resistant genes in CPR genomes.

For antimicrobial resistance profiling, we carried out an in-house BLAST search against the protein databases from ARG-ANNOT (Antibiotic Resistance Gene-ANNOTation) (42), BLDB (Beta-lactamase database) (52), and NDARO (National Database of Antibiotic Resistant Organisms) (53) containing 2,038, 4,260, and 5,735 sequences, respectively. To get a comprehensive view of the CPR resistome, we used relaxed parameters including a minimum percent identity and coverage length equal to 20% and 40%, respectively, and a maximum E value of 0.0001. All results were checked manually to remove duplications (Fig. 1).

Predicted AR genes in each CPR genome were individually compared to proteins in each AR database by reciprocal BLASTP (54). The number of reciprocal best hits was counted using an expectation value (E) of 0.0001 as the stringency threshold for determining a valid best hit (Fig. 1). Only the CPR protein sequence resulting from the reciprocal BLASTp and matched with the same AR gene resulting from the first BLASTp was conserved for the next step as the preliminary results of AR genes (Fig. 1).

To eliminate false-positive hits, a BLASTp search of the preliminary AR genes as a query data set was performed against the conserved domain database (CDD) (https://www.ncbi.nlm.nih.gov/Structure/cdd/wrpsb.cgi) (Fig. 1). The predicted AR genes with a protein domain necessary for the AR mechanism were subsequently selected. A literature review was conducted for each family of antibiotics detected in the CPR genomes to determine the mechanism of AR. We were interested only in the genes in which the AR mechanism depends on enzymatic activity and did not consider the mechanisms that require a further search for site mutations (Fig. 1).

AR-like genes detected in CPR tested genomes are represented using Cytoscape v.3.8.2 to highlight the link between different antibiotic families and distinct CPR superphyla. These genes are also represented in a multi-informative heat map created by the Displayr online tool (www.displayr.com), to show the distribution of different AR-like genes on CPR superphyla and their mechanisms of AR.

## References

[1] Castelle CJ, Banfield JF. 2018. Major new microbial groups expand diversity and alter our understanding of the tree of life. Cell 172:1181–1197. doi:10.1016/j.cell.2018.02.016. 29522741

[2] Ibrahim A, Colson P, Merhej V, Zgheib R, Maatouk M, Naud S, Bittar F, Raoult D. 2021. Rhizomal reclassification of living organisms. Int J Mol Sci 22:5643. doi:10.3390/ijms22115643. 34073251 PMC8199106

[3] Parks DH, Rinke C, Chuvochina M, Chaumeil P-A, Woodcroft BJ, Evans PN, Hugenholtz P, Tyson GW. 2017. Recovery of nearly 8,000 metagenome-assembled genomes substantially expands the tree of life. Nat Microbiol 2:1533–1542. doi:10.1038/s41564-017-0012-7. 28894102

[4] Brown CT, Hug LA, Thomas BC, Sharon I, Castelle CJ, Singh A, Wilkins MJ, Wrighton KC, Williams KH, Banfield JF. 2015. Unusual biology across a group comprising more than 15% of domain Bacteria. Nature 523:208–211. doi:10.1038/nature14486. 26083755

[5] Hug LA, Baker BJ, Anantharaman K, Brown CT, Probst AJ, Castelle CJ, Butterfield CN, Hernsdorf AW, Amano Y, Ise K, Suzuki Y, Dudek N, Relman DA, Finstad KM, Amundson R, Thomas BC, Banfield JF. 2016. A new view of the tree of life. Nat Microbiol 1:16048. doi:10.1038/nmicrobiol.2016.48. 27572647

[6] Wrighton KC, Thomas BC, Sharon I, Miller CS, Castelle CJ, VerBerkmoes NC, Wilkins MJ, Hettich RL, Lipton MS, Williams KH, Long PE, Banfield JF. 2012. Fermentation, hydrogen, and sulfur metabolism in multiple uncultivated bacterial phyla. Science 337:1661–1665. doi:10.1126/science.1224041. 23019650

[7] Kantor RS, Wrighton KC, Handley KM, Sharon I, Hug LA, Castelle CJ, Thomas BC, Banfield JF. 2013. Small genomes and sparse metabolisms of sediment-associated bacteria from four candidate phyla. mBio 4:e00708-13. doi:10.1128/mBio.00708-13. 24149512 PMC3812714

[8] Luef B, Frischkorn KR, Wrighton KC, Holman H-YN, Birarda G, Thomas BC, Singh A, Williams KH, Siegerist CE, Tringe SG, Downing KH, Comolli LR, Banfield JF. 2015. Diverse uncultivated ultra-small bacterial cells in groundwater. Nat Commun 6:6372. doi:10.1038/ncomms7372. 25721682

[9] Méheust R, Burstein D, Castelle CJ, Banfield JF. 2019. The distinction of CPR bacteria from other bacteria based on protein family content. Nat Commun 10:4173. doi:10.1038/s41467-019-12171-z. 31519891 PMC6744442

[10] Bernard C, Lannes R, Li Y, Bapteste É, Lopez P. 2020. Rich repertoire of quorum sensing protein coding sequences in CPR and DPANN associated with interspecies and interkingdom communication. mSystems 5:e00414-20. doi:10.1128/mSystems.00414-20. 33051376 PMC7567580

[11] Jaffe AL, Thomas AD, He C, Keren R, Valentin-Alvarado LE, Munk P, Bouma-Gregson K, Farag IF, Amano Y, Sachdeva R, West PT, Banfield JF. 2021. Patterns of gene content and co-occurrence constrain the evolutionary path toward animal association in candidate phyla radiation bacteria. mBio 12:e00521-21. doi:10.1128/mBio.00521-21. PMC8406219 34253055

[12] Rinke C, Schwientek P, Sczyrba A, Ivanova NN, Anderson IJ, Cheng J-F, Darling A, Malfatti S, Swan BK, Gies EA, Dodsworth JA, Hedlund BP, Tsiamis G, Sievert SM, Liu W-T, Eisen JA, Hallam SJ, Kyrpides NC, Stepanauskas R, Rubin EM, Hugenholtz P, Woyke T. 2013. Insights into the phylogeny and coding potential of microbial dark matter. Nature 499:431–437. doi:10.1038/nature12352. 23851394

[13] Dewhirst FE, Chen T, Izard J, Paster BJ, Tanner ACR, Yu W-H, Lakshmanan A, Wade WG. 2010. The human oral microbiome. J Bacteriol 192:5002–5017. doi:10.1128/JB.00542-10. 20656903 PMC2944498

[14] Tian R, Ning D, He Z, Zhang P, Spencer SJ, Gao S, Shi W, Wu L, Zhang Y, Yang Y, Adams BG, Rocha AM, Detienne BL, Lowe KA, Joyner DC, Klingeman DM, Arkin AP, Fields MW, Hazen TC, Stahl DA, Alm EJ, Zhou J. 2020. Small and mighty: adaptation of superphylum Patescibacteria to groundwater environment drives their genome simplicity. Microbiome 8:51. doi:10.1186/s40168-020-00825-w. 32252814 PMC7137472

[15] He X, McLean JS, Edlund A, Yooseph S, Hall AP, Liu S-Y, Dorrestein PC, Esquenazi E, Hunter RC, Cheng G, Nelson KE, Lux R, Shi W. 2015. Cultivation of a human-associated TM7 phylotype reveals a reduced genome and epibiotic parasitic lifestyle. Proc Natl Acad Sci USA 112:244–249. doi:10.1073/pnas.1419038112. 25535390 PMC4291631

[16] Murugkar PP, Collins AJ, Chen T, Dewhirst FE. 2020. Isolation and cultivation of candidate phyla radiation Saccharibacteria (TM7) bacteria in coculture with bacterial hosts. J Oral Microbiol 12:1814666. doi:10.1080/20002297.2020.1814666. 33209205 PMC7651992

[17] Ibrahim A, Maatouk M, Rajaonison A, Zgheib R, Haddad G, Bou-Khalil J, Raoult D, Bittar F. 2021. Adapted protocol for *Saccharibacteria* co-cultivation: two new members join the club of Candidate Phyla radiation. bioRxiv https://www.biorxiv.org/content/10.1101/2021.07.23.453610v1?rss=1. 10.1128/spectrum.01069-21 PMC8694215 35007432

[18] Bik EM, Long CD, Armitage GC, Loomer P, Emerson J, Mongodin EF, Nelson KE, Gill SR, Fraser-Liggett CM, Relman DA. 2010. Bacterial diversity in the oral cavity of 10 healthy individuals. ISME J 4:962–974. doi:10.1038/ismej.2010.30. 20336157 PMC2941673

[19] Fredricks DN, Fiedler TL, Marrazzo JM. 2005. Molecular identification of bacteria associated with bacterial vaginosis. N Engl J Med 353:1899–1911. doi:10.1056/NEJMoa043802. 16267321

[20] Grice EA, Segre JA. 2011. The skin microbiome. Nat Rev Microbiol 9:244–253. doi:10.1038/nrmicro2537. 21407241 PMC3535073

[21] Kuehbacher T, Rehman A, Lepage P, Hellmig S, Fölsch UR, Schreiber S, Ott SJ. 2008. Intestinal TM7 bacterial phylogenies in active inflammatory bowel disease. J Med Microbiol 57:1569–1576. doi:10.1099/jmm.0.47719-0. 19018031

[22] Abou Chacra L, Fenollar F. 2021. Exploring the global vaginal microbiome and its impact on human health. Microb Pathog 160:105172. doi:10.1016/j.micpath.2021.105172. 34500016

[23] Starr EP, Shi S, Blazewicz SJ, Probst AJ, Herman DJ, Firestone MK, Banfield JF. 2018. Stable isotope informed genome-resolved metagenomics reveals that Saccharibacteria utilize microbially-processed plant-derived carbon. Microbiome 6:122. doi:10.1186/s40168-018-0499-z. 29970182 PMC6031116

[24] Castelle CJ, Brown CT, Anantharaman K, Probst AJ, Huang RH, Banfield JF. 2018. Biosynthetic capacity, metabolic variety and unusual biology in the CPR and DPANN radiations. Nat Rev Microbiol 16:629–645. doi:10.1038/s41579-018-0076-2. 30181663

[25] Hugenholtz P, Tyson GW, Webb RI, Wagner AM, Blackall LL. 2001. Investigation of candidate division TM7, a recently recognized major lineage of the domain Bacteria with no known pure-culture representatives. Appl Environ Microbiol 67:411–419. doi:10.1128/AEM.67.1.411-419.2001. 11133473 PMC92593

[26] Danczak RE, Johnston MD, Kenah C, Slattery M, Wrighton KC, Wilkins MJ. 2017. Members of the Candidate Phyla Radiation are functionally differentiated by carbon- and nitrogen-cycling capabilities. Microbiome 5:112. doi:10.1186/s40168-017-0331-1. 28865481 PMC5581439

[27] Orsi WD, Richards TA, Francis WR. 2018. Predicted microbial secretomes and their target substrates in marine sediment. Nat Microbiol 3:32–37. doi:10.1038/s41564-017-0047-9. 29062087

[28] Anantharaman K, Brown CT, Hug LA, Sharon I, Castelle CJ, Probst AJ, Thomas BC, Singh A, Wilkins MJ, Karaoz U, Brodie EL, Williams KH, Hubbard SS, Banfield JF. 2016. Thousands of microbial genomes shed light on interconnected biogeochemical processes in an aquifer system. Nat Commun 7:13219. doi:10.1038/ncomms13219. 27774985 PMC5079060

[29] Weyrich LS, Duchene S, Soubrier J, Arriola L, Llamas B, Breen J, Morris AG, Alt KW, Caramelli D, Dresely V, Farrell M, Farrer AG, Francken M, Gully N, Haak W, Hardy K, Harvati K, Held P, Holmes EC, Kaidonis J, Lalueza-Fox C, de la Rasilla M, Rosas A, Semal P, Soltysiak A, Townsend G, Usai D, Wahl J, Huson DH, Dobney K, Cooper A. 2017. Neanderthal behaviour, diet, and disease inferred from ancient DNA in dental calculus. Nature 544:357–361. doi:10.1038/nature21674. 28273061

[30] Riesenfeld CS, Goodman RM, Handelsman J. 2004. Uncultured soil bacteria are a reservoir of new antibiotic resistance genes. Environ Microbiol 6:981–989. doi:10.1111/j.1462-2920.2004.00664.x. 15305923

[31] Martinez JL. 2008. Antibiotics and antibiotic resistance genes in natural environments. Science 321:365–367. doi:10.1126/science.1159483. 18635792

[32] D’Costa VM, King CE, Kalan L, Morar M, Sung WWL, Schwarz C, Froese D, Zazula G, Calmels F, Debruyne R, Golding GB, Poinar HN, Wright GD. 2011. Antibiotic resistance is ancient. Nature 477:457–461. doi:10.1038/nature10388. 21881561

[33] Blair JMA, Webber MA, Baylay AJ, Ogbolu DO, Piddock LJV. 2015. Molecular mechanisms of antibiotic resistance. Nat Rev Microbiol 13:42–51. doi:10.1038/nrmicro3380. 25435309

[34] Perkins HR, Nieto M. 1974. The chemical basis for the action of the vancomycin group of antibiotics. Ann N Y Acad Sci 235:348–363. doi:10.1111/j.1749-6632.1974.tb43276.x. 4369274

[35] Stogios PJ, Savchenko A. 2020. Molecular mechanisms of vancomycin resistance. Protein Sci 29:654–669. doi:10.1002/pro.3819. 31899563 PMC7020976

[36] Colson P, Pinault L, Azza S, Armstrong N, Chabriere E, La Scola B, Pontarotti P, Raoult D. 2020. A protein of the metallo-hydrolase/oxidoreductase superfamily with both beta-lactamase and ribonuclease activity is linked with translation in giant viruses. Sci Rep 10:21685. doi:10.1038/s41598-020-78658-8. 33303919 PMC7729979

[37] Tooke CL, Hinchliffe P, Bragginton EC, Colenso CK, Hirvonen VHA, Takebayashi Y, Spencer J. 2019. β-Lactamases and β-lactamase inhibitors in the 21st century. J Mol Biol 431:3472–3500. doi:10.1016/j.jmb.2019.04.002. 30959050 PMC6723624

[38] Leclercq R. 2002. Mechanisms of resistance to macrolides and lincosamides: nature of the resistance elements and their clinical implications. Clin Infect Dis 34:482–492. doi:10.1086/324626. 11797175

[39] Poehlsgaard J, Douthwaite S. 2003. Macrolide antibiotic interaction and resistance on the bacterial ribosome. Curr Opin Invest Drugs 4:140–148. 12669373

[40] Zhanel GG, Dueck M, Hoban DJ, Vercaigne LM, Embil JM, Gin AS, Karlowsky JA. 2001. Review of macrolides and ketolides: focus on respiratory tract infections. Drugs 61:443–498. doi:10.2165/00003495-200161040-00003. 11324679

[41] Jana S, Deb JK. 2006. Molecular understanding of aminoglycoside action and resistance. Appl Microbiol Biotechnol 70:140–150. doi:10.1007/s00253-005-0279-0. 16391922

[42] Gupta SK, Padmanabhan BR, Diene SM, Lopez-Rojas R, Kempf M, Landraud L, Rolain J-M. 2014. ARG-ANNOT, a new bioinformatic tool to discover antibiotic resistance genes in bacterial genomes. Antimicrob Agents Chemother 58:212–220. doi:10.1128/AAC.01310-13. 24145532 PMC3910750

[43] Illergård K, Ardell DH, Elofsson A. 2009. Structure is three to ten times more conserved than sequence—a study of structural response in protein cores. Proteins 77:499–508. doi:10.1002/prot.22458. 19507241

[44] Ruiz-Perez CA, Conrad RE, Konstantinidis KT. 2021. MicrobeAnnotator: a user-friendly, comprehensive functional annotation pipeline for microbial genomes. BMC Bioinformatics 22:11. doi:10.1186/s12859-020-03940-5. 33407081 PMC7789693

[45] Khabthani S, Hamel M, Baron SA, Diene SM, Rolain J-M, Merhej V. 2021. fosM, a new family of fosfomycin resistance genes identified in bacterial species isolated from human microbiota. Antimicrob Agents Chemother 65:e01712-20. doi:10.1128/AAC.01712-20. 33199384 PMC7848996

[46] Poole K. 2005. Efflux-mediated antimicrobial resistance. J Antimicrob Chemother 56:20–51. doi:10.1093/jac/dki171. 15914491

[47] Lewis K. 2013. Platforms for antibiotic discovery. 5. Nat Rev Drug Discov 12:371–387. doi:10.1038/nrd3975. 23629505

[48] Bush K. 2018. Past and present perspectives on β-lactamases. Antimicrob Agents Chemother 62:e01076-18. doi:10.1128/AAC.01076-18. 30061284 PMC6153792

[49] Diene SM, Pinault L, Keshri V, Armstrong N, Khelaifia S, Chabrière E, Caetano-Anolles G, Colson P, La Scola B, Rolain J-M, Pontarotti P, Raoult D. 2019. Human metallo-β-lactamase enzymes degrade penicillin. Sci Rep 9:12173. doi:10.1038/s41598-019-48723-y. 31434986 PMC6704141

[50] Diene SM, Pinault L, Armstrong N, Azza S, Keshri V, Khelaifia S, Chabrière E, Caetano-Anolles G, Rolain J-M, Pontarotti P, Raoult D. 2020. Dual RNase and β-lactamase activity of a single enzyme encoded in archaea. Life 10:280. doi:10.3390/life10110280. PMC7697635 33202677

[51] Brettin T, Davis JJ, Disz T, Edwards RA, Gerdes S, Olsen GJ, Olson R, Overbeek R, Parrello B, Pusch GD, Shukla M, Thomason JA, Stevens R, Vonstein V, Wattam AR, Xia F. 2015. RASTtk: a modular and extensible implementation of the RAST algorithm for building custom annotation pipelines and annotating batches of genomes. Sci Rep 5:8365. doi:10.1038/srep08365. 25666585 PMC4322359

[52] Naas T, Oueslati S, Bonnin RA, Dabos ML, Zavala A, Dortet L, Retailleau P, Iorga BI. 2017. Beta-lactamase database (BLDB) – structure and function. J Enzyme Inhib Med Chem 32:917–919. doi:10.1080/14756366.2017.1344235. 28719998 PMC6445328

[53] National Center for Biotechnology Information. 2016. Bacterial antimicrobial resistance reference gene database. BioProject ID 31304. https://www.ncbi.nlm.nih.gov/bioproject/313047.

[54] Altschul SF, Madden TL, Schäffer AA, Zhang J, Zhang Z, Miller W, Lipman DJ. 1997. Gapped BLAST and PSI-BLAST: a new generation of protein database search programs. Nucleic Acids Res 25:3389–3402. doi:10.1093/nar/25.17.3389. 9254694 PMC146917

