# Supplementary figures and images for: Small and Equipped: the Rich Repertoire of Antibiotic Resistance Genes in Candidate Phyla Radiation Genomes
Source: mSystems. 2021 Dec 7;6(6):e00898-21. doi: 10.1128/mSystems.00898-21 (PMC8651080; doi:10.1128/mSystems.00898-21)

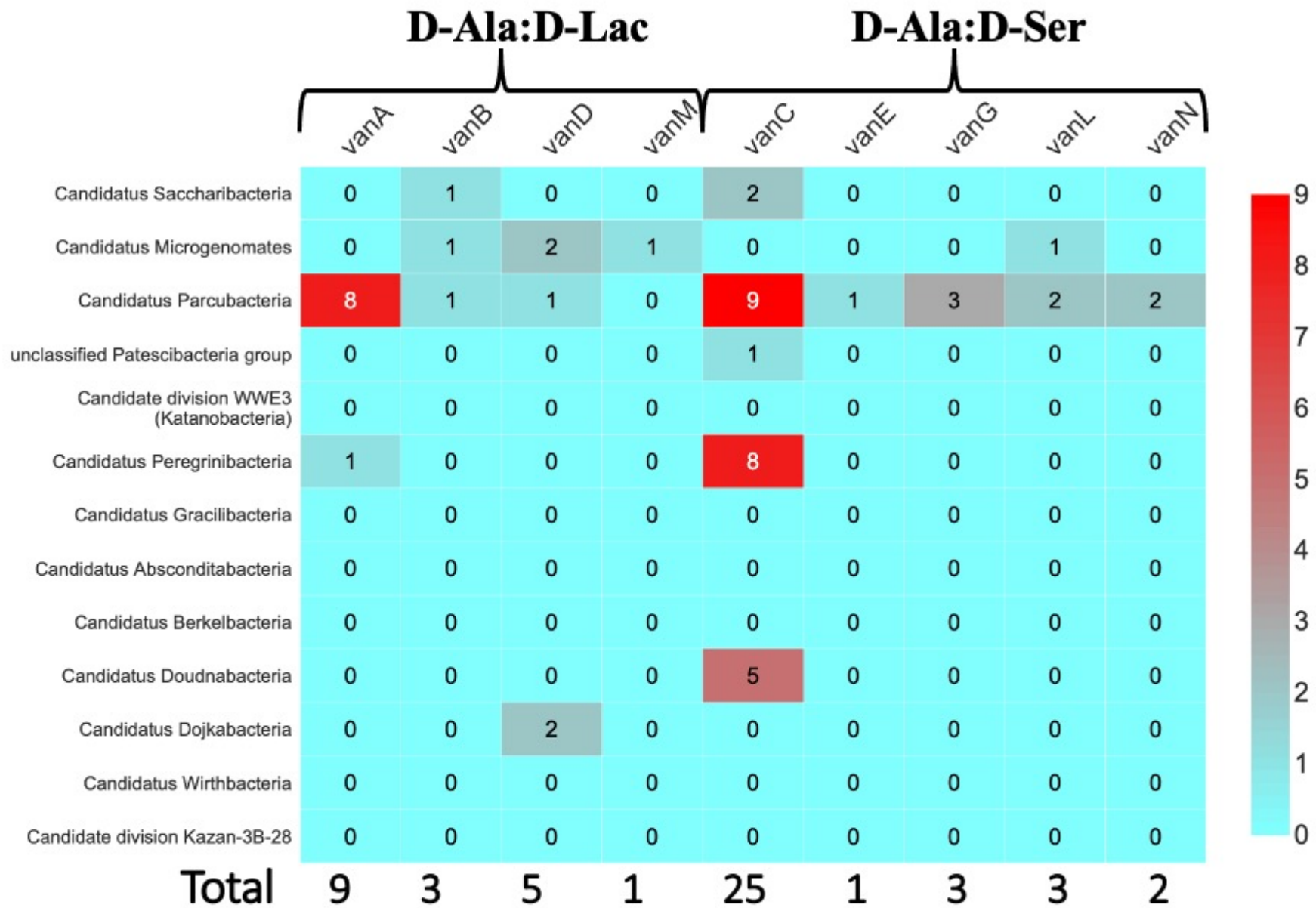

Supplement: FIG S1 [file msystems.00898-21-sf001.pdf]

# Antibiotic families

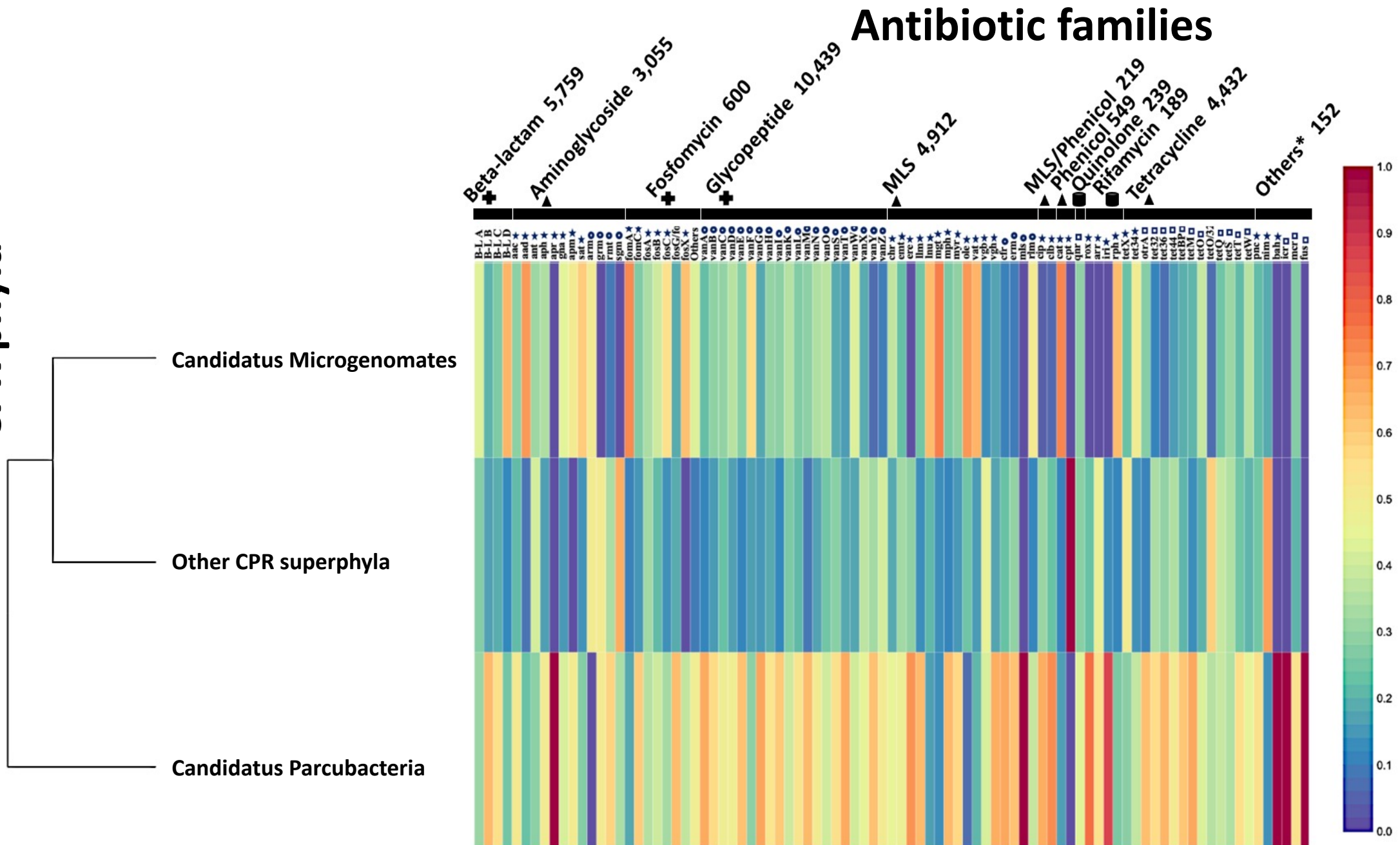

Supplement: FIG S2 [file msystems.00898-21-sf002.pdf]

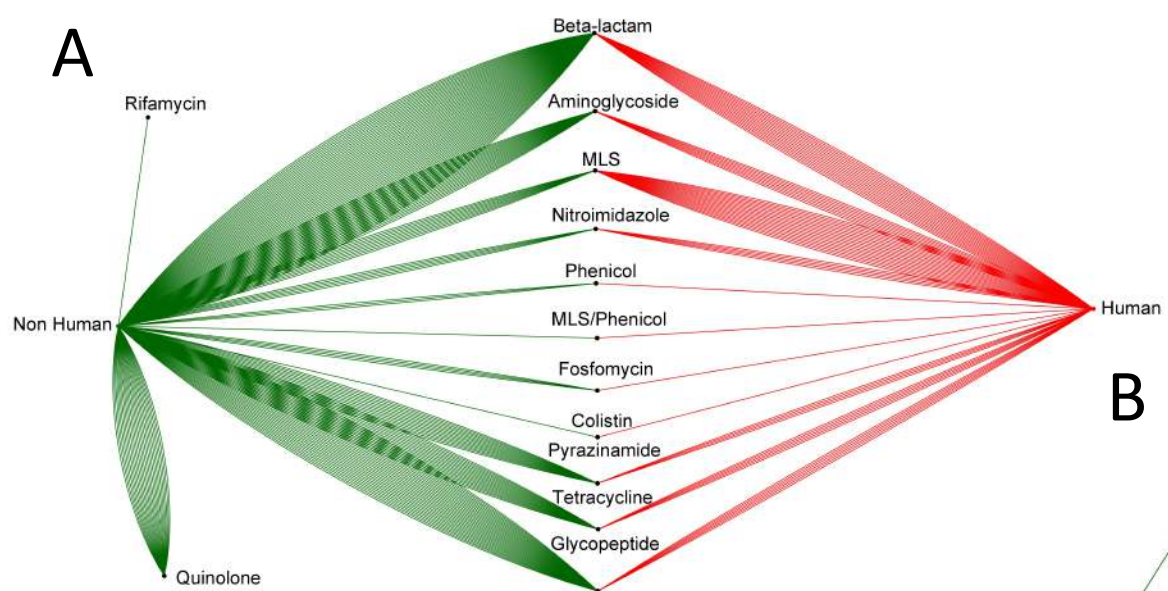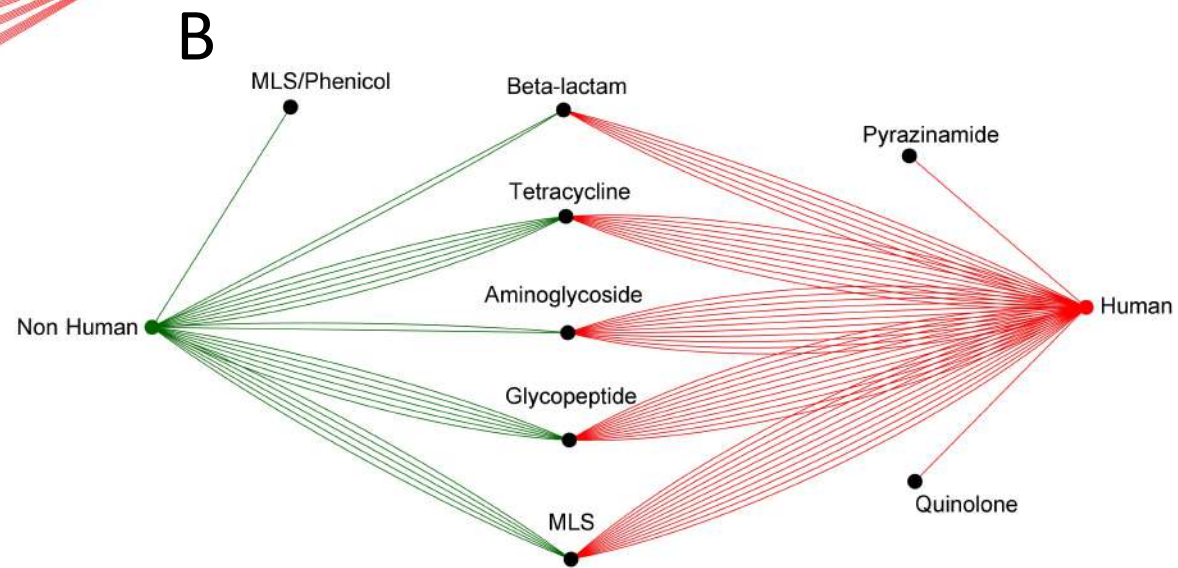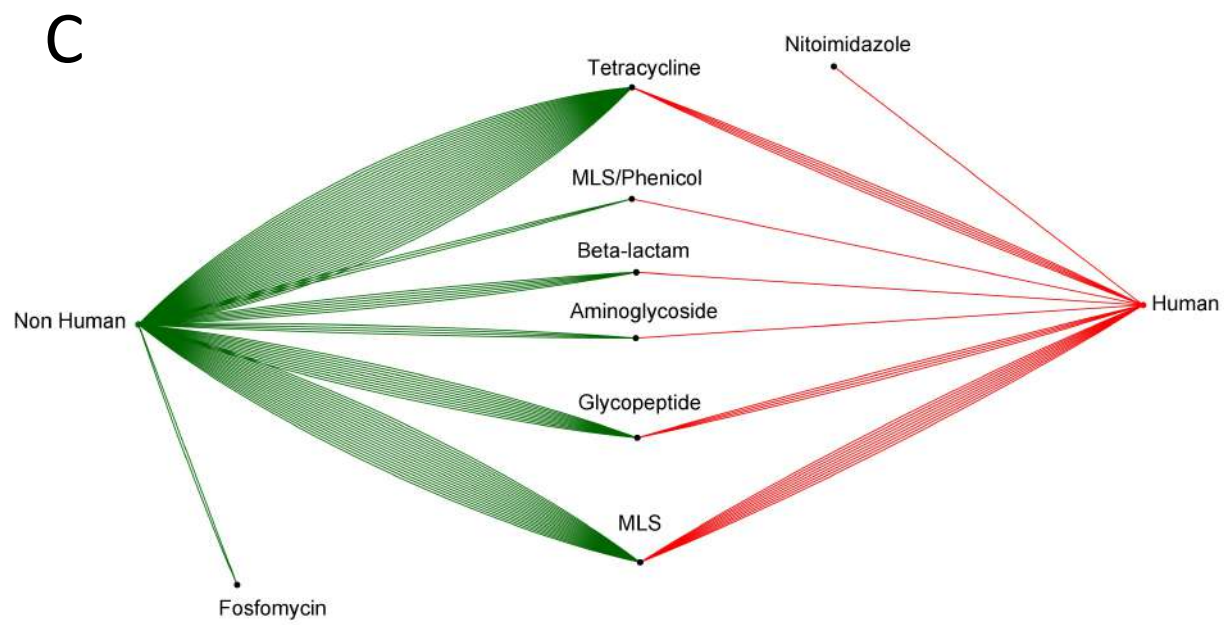

Supplement: FIG S3 [file msystems.00898-21-sf003.pdf]

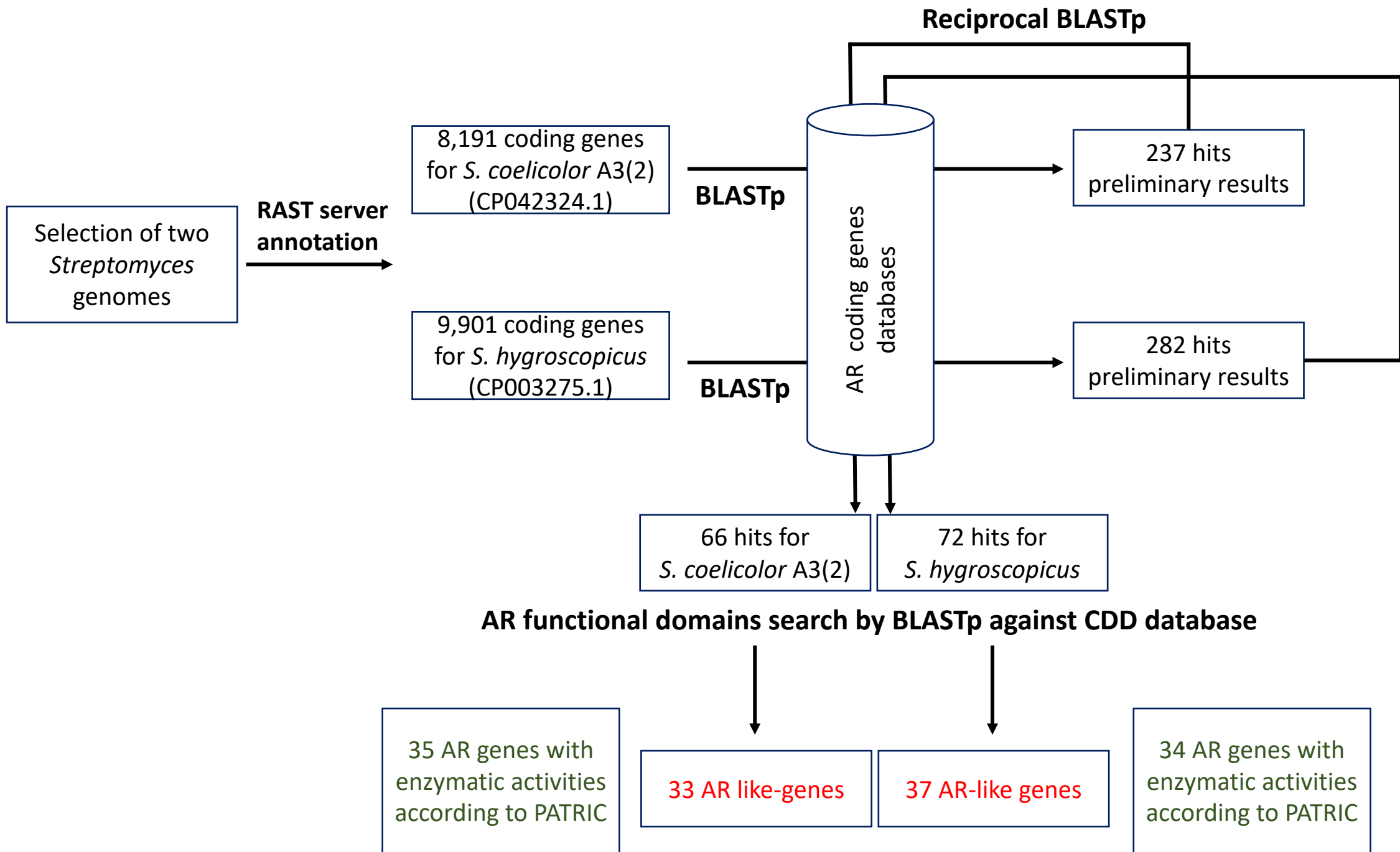

Supplement: FIG S4 [file msystems.00898-21-sf004.pdf]
